# Prevalence of Blood-Borne Viruses and Predictors of Risk in Potential Organ Donors in Australia

**DOI:** 10.3389/ti.2022.10395

**Published:** 2022-05-03

**Authors:** Martin J. Dutch, Cameron J. Patrick, Peter A. Boan, Jonathan C. Knott, Helen I. Opdam

**Affiliations:** ^1^ Royal Melbourne Hospital, Melbourne, VIC, Australia; ^2^ DonateLife (Victoria), Melbourne, VIC, Australia; ^3^ Department of Critical Care, University of Melbourne, Melbourne, VIC, Australia; ^4^ Statistical Consultancy Unit, University of Melbourne, Melbourne, VIC, Australia; ^5^ Microbiology Department, PathWest Laboratory Medicine, Perth, WA, Australia; ^6^ Department of Infectious Disease, Fiona Stanley Hospital, Perth, WA, Australia; ^7^ Emergency Department, Royal Melbourne Hospital, Melbourne, VIC, Australia; ^8^ Intensive Care Unit, Austin Hospital, Melbourne, VIC, Australia; ^9^ Australian Organ and Tissue Authority, Canberra, ACT, Australia

**Keywords:** risk, organ donation, predictive value, behavior, disease transmission, Australia, questionnaire, residual risk

## Abstract

Internationally, the designation of a patient as an increased viral risk organ donor has been associated with lower utilisation rates. The actual prevalence of blood borne viruses in Australian potential organ donors, and the predictive performance of questionnaires administered to stratify this risk, remains unknown. We conducted a retrospective review of all patients who commenced workup for donation on the national database between 2014–2020. The prevalence of HIV, Active HBV and Active HCV in 3650 potential organ donors was 0.16%, 0.9%, and 2.2%, respectively. The behavioural risk profile was assessed in a subset of 3633 patients. Next-of-kin reported increased risk behaviours were associated with an increased prevalence of HCV but not of HIV or HBV (OR 13.8, *p* < 0.01, OR 0.3. *p* = 0.42, OR 1.5, *p* = 0.14). Furthermore, the majority of HIV and HBV infections occurred in potential donors without a disclosed history of increased risk behaviours. In this series, donors had a higher prevalence of HCV, and similar rates of HBV and HIV to the broader community. Behavioural transmission risks were poorly predictive of HIV and HBV. Rather than pre-transplantation behavioural risk screening, routine post-transplant recipient screening may provide a more powerful tool in mitigating the consequences of unexpected viral transmission.

## Graphical Abstract

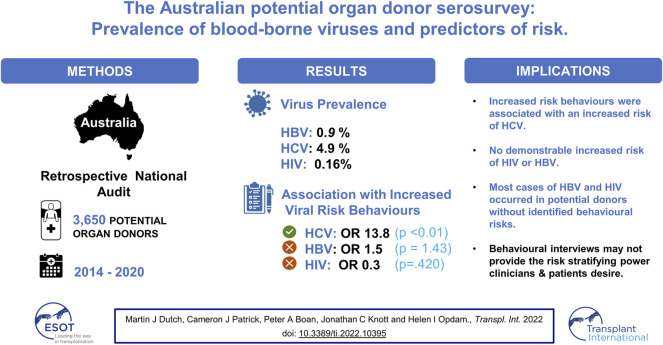

## Introduction

The potential for donor derived infections of human immunodeficiency virus (HIV), hepatitis B virus (HBV) and hepatitis C virus (HCV) is an important consideration in the medical suitability assessment of any organ donor. In addition to routine pathology screening, the structured exploration of donor increase-viral-risk behaviours (IRBs) is a routine component in the assessment of risk for transmission of blood-borne viruses (BBVs) (1).

Conducting a structured behavioural interview is a significant undertaking. The potential donor has often died in a sudden and unexpected manner and acutely bereaved family members are requested to engage in an extensive screening interview of their relative’s medical history and behaviours. The Australian interview contains over 40 questions and covers a variety of sensitive subjects including the deceased’s sexual health, illicit drug use, forensic and psychiatric histories.

The identification of what constitutes an increased-viral risk behaviour (IRB) has historically been derived from a combination of discerning biologically plausible mechanisms for transmission, self-reported behaviours in ecological studies and expert opinion (2).

Potential donors who have no evidence of BBV exposure on blood testing but are thought to have engaged in recent IRBs are designated as increased-viral-risk donors (IVRDs). The underlying premise being engagement in recent IRBs is thought to produce a clinically meaningful elevation in the risk of window period infection when compared to standard risk organ donors.

Designation as an IVRD may have significant implications. International experience shows IVRD designation is associated with lower utilisation of organs (3, 4), resulting in less patients being transplanted. This is despite evidence that the objective risk of transmission is extremely low (5–7), and that IVRD organs come from donors who are on average younger, and have less comorbidities (8, 9). Recipients who accept an IVRD organ offer, have fewer post-transplant complications, and in some series, improved long-term survival (10–13).

A recent study, from New South Wales, Australia, highlighted that a significant portion of potential donors did not proceed to donation, based solely on the presence of increased risk behaviours (14). In some instances, the decision not to progress with donation workup occurred prior to pathology screening.

The prevalence of BBVs and IRBs in a national cohort of Australian potential organ donors has not been previously described. The external validity of IRBs derived from US populations has also not been tested in an Australian context (15).

Given the effort required to elicit a history of behavioural risks, and the sequelae of a designation of increased risk, it is important to confirm that currently utilised questions successfully risk stratify potential organ donors.

This study aims to determine the prevalence of BBVs within potential organ donors in Australia and determine the utility of currently used behavioural questions to differentiate risk within this cohort.

## Patients and Methods

### Design

A retrospective audit of the national electronic donor record (EDR) database was undertaken to identify all potential organ donors referred over a 6-year period between March 2014 and March 2020. The database is hosted by the Australian Organ and Tissue Authority (AOTA).

The project was approved by the Melbourne Health human research ethics committee (QA2019030), the AOTA Data Governance Committee, and undertaken with the approval of each of the eight state and territory jurisdictions. The study was conducted in accordance with the ethical standards laid down in the Declarations of Helsinki and Istanbul.

### Setting

Australia is a multicultural nation with 30% of the population having been born overseas and 46% of Australians having at least one parent born overseas (16, 17). The prevalence of HBV is 0.9% (18), with most cases occurring in migrants from higher prevalence countries. The prevalence of HIV and anti-HCV are 0.1% (18) and 2.3% respectively (19).

The AOTA coordinates the DonateLife network, which includes the organ procurement entity in each state and territory, and a network of over 90 donation hospitals. In 2019, Australia had an estimated population of 25.6 million and a donation rate of 21.6 deceased organ donors per million population (20).

In partnership with AOTA, the Transplantation Society of Australia and New Zealand (TSANZ) author donor evaluation policy and issue national guidelines on the requirements for testing for BBV in potential organ donors (1). It is then up to individual transplant clinicians and patients to determine the risk benefit of an individual organ offer.

#### Testing for Blood-Borne Viruses

There has been an evolution in mandatory and recommended testing for BBV since 2014. From 2014 mandatory tests were HIV antibody, Hepatitis B surface antibody (HBsAb), Hepatitis B surface antigen (HBsAg), Hepatitis B core antibody (HBcAb) and Hepatitis C antibody (HCV Ab). Nucleic acid testing (NAT) for HCV and HIV was recommended for IVRDs. From April 2016, Hepatitis B NAT was also recommended for IVRDs (1). From May 2019 the testing requirements specified that the HIV serology testing be a combination antigen/antibody assay and also stated that prospective NAT for HIV, HBV and HCV was required wherever this was logistically feasible and was strongly advised for IVRDs. However, if serological screening results were negative, and awaiting NAT results would represent an unreasonable delay, transplantation could proceed at the discretion of the transplant team and with appropriate recipient consent (1). The majority of national deceased organ donor serology and NAT testing is undertaken by Australian Red Cross Lifeblood in dedicated state-based processing centres.

#### Administration of the Behavioural Risk Assessment Questionnaire

As part of the workup for donation, specialist donor coordinator nursing staff conduct interviews with family members and close associates of the potential organ donor. A behavioural risk assessment questionnaire (BRAQ) is utilised, with occasionally more than one administered if separate interviews are required according to family circumstances. The BRAQ includes more than 40 questions, and records respondent’s answers both dichotomously (yes/no), and with free text fields. Answers are recorded in the EDR.

### Study Population and Sampling

The target population for this study were patients who commenced workup for organ donation in Australia.

**FIGURE 1:**
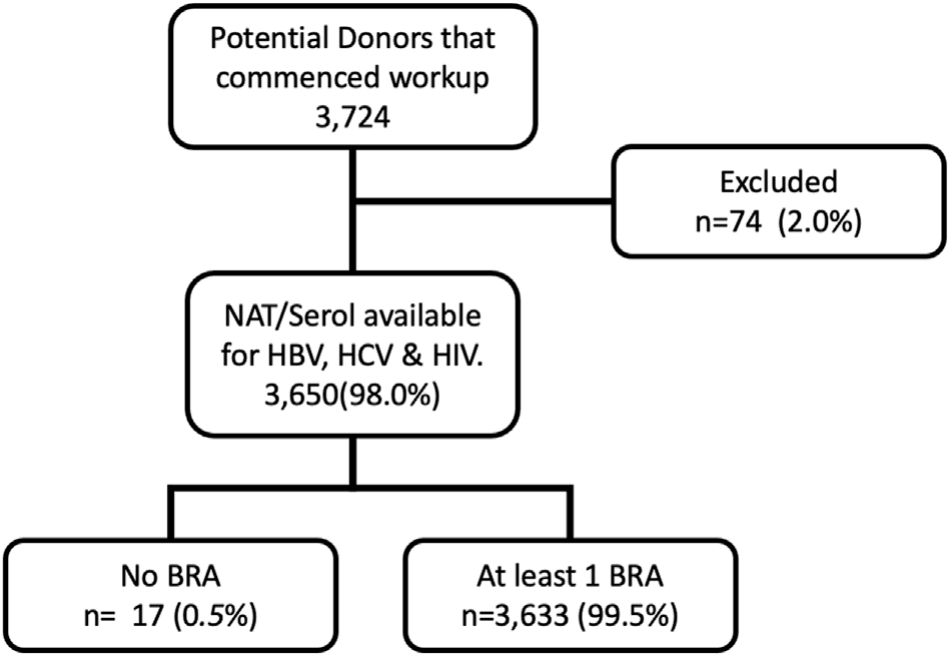
Study Flow Diagram 2. BRA (behavioural risk assessment questionnaire).

We included all patients who had an EDR commenced and excluded those who did not progress to BBV testing for all three viruses (Figure 1). EDR commencement occurred when provisional family consent was obtained and prior to the administration of the BRAQ or testing for BBVs. Patients were excluded if they did not progress to BBV testing or did not have a BRAQ administered.

### Data Collection and Classification of Cases

Basic demographic data, results of the BRAQ, and pathology results for HIV, HBV and HCV were extracted for analysis.

#### Blood-Borne Virus Exposure Status

Blood specimens were initially classified by their haemodilution status. Specialist donor coordinator nurses audited the administration of intravenous therapy and blood product transfusions received in the 48 h prior to blood sampling for BBV testing. Pathology specimens were classified as potentially haemodiluted if the volumes of crystalloids, colloids and blood products, as a percentage of total plasma and blood volumes, exceeded a prescribed threshold (Supplementary Figure S1).

For the purpose of this study, a case was classified as having an unknown viral status, when there was either:

1) No serology or NAT undertaken for the virus.

OR

2) All tests were undertaken on haemodiluted samples AND all sample results were negative for the virus.

A case was classified as “exposed” to a virus, when serology or NAT indicated either current or past infection, with one the following tests being positive: HIV serology, HIV NAT, HBcAb, HBsAg, HBV NAT, HCV Ab, or HCV NAT. “Active” infection was defined by one of the following tests being positive: HIV serology, HIV NAT, HBsAg, HBV NAT, or HCV NAT.

Inactive HBV infection may result in reactivation and clinically significant disease in liver transplant recipients (1). Inactive HBV was defined as any evidence of prior HBV exposure (HBcAb) but no evidence of active replication (HBsAg -ve, HBV NAT -ve).

Inactive HCV infection may result from spontaneous clearance or successful treatment (1). Inactive HCV was defined as evidence of previous HCV exposure (anti-HCV positive), with no detectable HCV RNA on NAT.

This classification held, even if the specimen was flagged as haemodiluted. In cases where the test was repeated and found to subsequently be negative, the case was still classified as exposed (Supplementary Table S4).

As such we have adopted a conservative case definition where an exposed case may indicate current infection, past infection or a false positive.

“Any exposure to BBV” was defined as a positive test result for exposure to any BBV, and “Any active BBV” was defined as positive test result for active BBV infection.

Hepatitis B immunity was defined as being HBsAb positive, with negative HBcAb, HBsAg and HBV NAT.

A case was classified negative for a virus when a valid, non-haemodiluted sample was analysed, and all NAT and acute and chronic serological markers were negative.

#### Presence of Increased-Viral-Risk Behaviours

Within Australia, patients must fulfill at least one of 11 criteria to be designated an IVRD. These criteria consist of eight IRBs, and an additional three clinical scenarios that may confer increased risk which are not included in our analysis:

1) Where the potential donor is already known to have a BBV
2) Where the medical and behavioural history cannot be obtained
3) When a non-haemodiluted blood specimen cannot be obtained

Eight IRBs were screened for during the administration of the BRAQ. They are:

1) Person who injects drugs (PWID) by intravenous, intramuscular, or subcutaneous route for non-medical reasons
2) Men who have sex with men (MSM)
3) People who have been in lockup, jail, prison, or a juvenile correctional facility for more than 72 consecutive hours
4) People who have had sex in exchange for money or drugs
5) People who have had sex with a person in any of the above groups
6) People who have been newly diagnosed with, or have been treated for, syphilis, gonorrhoea, chlamydia, or genital ulcers
7) A child who is 18 months old or younger and born to a mother known to be infected with, or at increased risk for, HIV, HBV or HCV infection
8) A child who has been breastfed within the preceding 6 months, and the mother is known to be infected with, or at increased risk for, HIV, HBV or HCV infection

At the commencement of the study period, these IRBs were recorded in the EDR if they occurred within the last 12 months, or in the case of injecting drug use, had ever occurred. In line with changes by the TSANZ, after April 2016, the database recorded these behaviours only if they occurred within the last 10 weeks. A composite variable “Any predictors” was utilised as the presence of at least one IRB designating an IVRD (Supplementary Tables S5–S7).

### Analysis

A priori 2-sided sample size calculations were undertaken (alpha 0.05, power 0.80). Modelling used the prevalence of BBVs in Australian deceased tissue donors (21) and examined a range of potential risk-factor prevalence (1–30%) (Supplementary Table S1).

Results demonstrate that for an expected case series of 3,288, the sample size would be suitable to reveal clinically significant increases (OR 10) in prevalence for HBV (for risk factors frequencies >1%) and HCV (risk factor frequencies ≥5%), but were likely to be underpowered to find associations with HIV for all but the most prevalent risk factors (Supplementary Tables S2, S3).

The composite IRB variable (“Any predictors”) was analysed for its prediction of “Any BBV exposure” through calculation of sensitivity, specificity, positive predictive value, negative predictive value and odds ratio.

Statistical analysis was performed using STATA/IC 15.1 (StataCorp LLC).

The prevalence of each disease and risk factor was determined. Statistical comparison of proportions was undertaken using Chi-square analysis with Fisher’s exact method (alpha 0.05). Difference between non-parametric variables were analysed using Wilcox rank-sum. Confidence intervals for proportions were calculated using the binomial exact method.

## Results

Between March 2014 and March 2020, 3,724 individuals were referred to DonateLife and had an EDR commenced. Seventy-four individuals had workup for donation terminated prior to testing for all three viruses. Typical reasons for discontinuation include medical instability, identification of a contraindication to donation, and withdrawal of consent to proceed (Figure 1).

In total, 3,650 patients underwent pathology testing for BBV exposure. A combination of both NAT and serology screening was undertaken in the vast majority of cases (89.5%). Serology testing without NAT occurred in 10.5% of patients for HIV, 9.8% of patients for HBV, and 9.7% of patients for HCV. Over the study period, the fraction of potential donors that underwent combined NAT and serology testing increased from 86% to 95%.

The average potential donor age was 51 years and they were more commonly male (59%), and were referred from all states and territories.

Approximately three in every four patients in this study proceeded to organ donation (Table 1). Of patients who did not proceed, IRBs or BBV exposure were more prevalent (IRB: Proceed 36.5% vs Did not proceed 40.4%, p 0.041, BBV: Proceed 3.6% vs Did not proceed: 13.8%, *p* < 0.001).

**TABLE 1:** Characteristics of Potential Organ Donors who have undertaken testing for blood borne viruses.

Demographics (*n* = 3,650)			
Age	51	IQR	(36–62)
Gender	n	%	
Male	2,141	58.7	
Female	1,509	41.3	
Donation Outcome	n	%	
Proceeded to donation	2,847	78.0	
Did not proceed to donation	803	22.0	
Virus Exposure	n	%	% CI
HIV	6	0.2	(0.1–0.4)
HBV			
Active	33	0.9	(0.6–1.2)
Inactive	181	5.0	(4.4–5.8)
Active and Inactive	214^a^	5.9	(5.1–6.7)
Vaccine Immunity^b^	1,061	30.9	(29.3–32.4)
HCV			
Active	73	2.2	(1.7–2.8)
Inactive	92	2.9	(2.3–3.5)
Active and Inactive	179^a^	4.9	(4.2–5.7)
At least 1			
Active Infection^c^	106	3.24	(2.6–3.9)
Any Exposure	344	9.4	(8.5–10.4)
Increased Risk Behaviours (*n* = 3,663)	n	%	
1. PWID	187	5.2	(4.5–5.9)
2. MSM	45	1.2	(0.9–1.7)
3. Detention	340	9.4	(8.4–10.4)
4. Sex Worker	23	0.6	(0.4–0.9)
5. Increased Risk Partner	1289	35.5	(33.9–37.1)
6. STI	81	2.2	(1.8–2.8)
7. Child (IRM)	0	0.0	(0.0–0.1)
8. Breastfed (IRM)	1	0.0	(0.0–0.2)
At least 1 IRB identified	1,365	37.6	(36.0–39.2)

HIV = human immunodeficiency virus; HBV Hepatitis B Virus, HCV Hepatitis C Virus. PWID, person who injects drugs; MSM, men who have sex with men, Detention = Admission to a lock up, prison or psychiatric facility, STI, sexually transmitted infection; IRM, Increased risk mother; IRB, increased risk behaviour.

a Includes serologically positive patients who did not have NAT testing. Not the sum of active and inactive cases.

^b^HbSAb, in absence of HBcAb or other markers. Available in 3,438 cases.

c Includes all HIV exposed patients, NAT+ve for HCV or HBV patients, and those with HBsAg.

The majority of patients who failed to proceed to donation were being considered for donation via the donation-after-circulatory-death pathway (74%). Death not occurring within the time period required for successful donation and transplantation has previously been shown to be a common reason for failure of donation to proceed in these patients (22).

In the study cohort of 3,650 patients who had undergone pathology testing 99.5% of potential donors who had BBV testing had at least one BRAQ administered (see diagram 1). In some cases, more than one questionnaire was administered.

### Blood-Borne Virus Exposure Prevalence

Nearly ten percent of potential donors who commenced workup for organ donation had evidence of exposure to a BBV. Exposure to HBV was the most prevalent (5.85%), followed by HCV (4.98%), then HIV (0.16%).

In total, 106 (3.24%) potential donors had active infection with a BBV.

A sizable proportion of patients with a BBV exposure were co-infected. Of the 214 patients with either active or inactive HBV, 50 (23%) had HCV co-exposure. Two patients had exposure to all three viruses.

The majority of HBV infections were inactive with active infection occurring in less than 1% of potential donors. There was serological evidence of previous vaccination in 30% of potential donors.

### Prevalence of IVRBs

During the study period 4,009 BRAQs were administered to the families and associates of 3,633 potential organ donors.

Over one third of potential donors who commenced workup for organ donation had a history of engaging in one or more IRBs (Table 1).

The most commonly identified IRBs were having a sexual relationship with an IRB partner (35%), followed by a history of being in detention in a lockup, jail, prison, or a juvenile correctional facility (9%) (Table 1).

Potential donors with IRBs were a median of 13 years younger than those without IRBs, were more likely to be male (68%), and more likely to have evidence of BBV exposure and less likely to proceed to donation (Table 2).

**TABLE 2:** Comparison of patient with and without increased risk behaviours (*n* = 3,663).

		IRB n = 1,365 (37.6%)	No IRB n = 2268 (62.4%)	p
Age	median (IQR)	43 (31–53)	56 (43–65)	<0.001
Gender: Male	n (%)	927 (67.9)	1,199 (52.9)	<0.001
BBV exposure	n (%)	204 (15.0)	136 (6.0)	<0.001
Proceeded to Organ Donation	n (%)	1,040 (76.2)	1,805 (79.6)	0.016

IRB, Increased risk behaviour; BBV, Blood-borne virus; BRAQ, behavioural risk assessment questionnaire.

### Increased-Viral Risk Behaviours Associated With Blood-Borne Virus Exposure

Only six potential donors with HIV were referred during the study period (Table 1). None of the IRBs were associated with a significantly increased prevalence of HIV over the study period.

While inactive HBV was shown to have a higher prevalence in both PWID and persons who engaged in sex work, only injecting drug use was associated with a higher prevalence of active HBV (PWID 4.81% vs non-PWID 0.67%, OR 7.56, *p* < 0.001).

Several IRBs were associated with exposure to HCV (Table 3). These included being a PWID, being in detention, sex work, being a MSM or having a sexual partner of any of the preceding groups.

**TABLE 3:** Frequency of blood-borne viruses in potential donors with increased risk behaviours.

Increased risk behaviour	HIV			Active HBV			Inactive HBV			Active HCV			Inactive HCV		
	Cases in patients with IRB	Cases in patients without IRB	p	Cases in patients with IRB	Cases in patients without IRB	p	Cases in patients with IRB	Cases in patients without IRB	p	Cases in patients with IRB	Cases in patients without IRB	p	Cases in patients with IRB	Cases in patients without IRB	p
	n/total (%)	n/total (%)		n/total (%)	n/total (%)		n/total (%)	n/total (%)		n/total (%)	n/total (%)		n/total (%)	n/total (%)	
PWID	1/187 (0.53)	5/3446 (0.15)	0.272	9/187 (4.81)	23/3446 (0.67)	<0.001	40/188 (21.28)	141/3469 (4.06)	<0.001	37/166 (22.29)	35/3127 (1.12)	<0.001	67/129 (51.94)	25/3070 (0.81)	<0.001
MSM	1/45 (2.22)	5/3588 (0.14)	0.072	0/45 (0.00)	32/3588 (0.89)	1.000	5/45 (11.11)	176/3612 (4.87)	0.069	1/43 (2.33)	71/3250 (2.18)	0.616	5/41 (12.2)	87/3158 (2.75)	0.006
Detention	0/340 (0.00)	6/3293 (0.18)	1.000	3/340 (0.88)	29/3293 (0.88)	1.000	18/341 (5.28)	163/3316 (4.92)	0.793	23/316 (7.28)	49/2977 (1.65)	<0.001	40/292 (13.7)	52/2907 (1.79)	<0.001
Sex Worker	0/23 (0.00)	6/3610 (0.17)	1.000	0/23 (0.00)	32/3610 (0.89)	1.000	6/23 (26.09)	175/3634 (4.82)	<0.001	1/22 (4.55)	71/3271 (2.17)	0.386	4/22 (18.18)	88/3177 (2.77)	0.003
Increased Risk Partner	1/1289 (0.08)	5/2344 (0.21)	0.432	11/1289 (0.85)	21/2344 (0.90)	1.000	64/1291 (4.96)	117/2366 (4.95)	1.000	57/1165 (4.89)	15/2128 (0.7)	<0.001	81/1101 (7.36)	11/2098 (0.52)	<0.001
STI	0/81 (0.00)	6/3552 (0.17)	1.000	0/81 (0.00)	32/3552 (0.90)	1.000	3/81 (3.70)	178/3576 (4.98)	0.797	3/68 (4.41)	69/3225 (2.14)	0.185	0/64 (0)	92/3135 (2.93)	0.262
Breastfed (IRM)	0/1 (0.00)	6/3632 (0.17)	1.000	0/1 (0.00)	32/3632 (0.88)	1.000	0/1 (0.00)	181/3656 (4.95)	1.000	0/1 (0)	72/3292 (2.19)	1.000	0/1 (0)	92/3198 (2.88)	1.000
Any IRB	1/1366 (0.07)	5/2284 (0.22)	0.421	16/1351 (1.17)	17/2273 (0.74)	0.207	74/1367 (5.41)	107/2290 (4.67)	0.344	59/1228 (4.8)	13/2065 (0.63)	<0.001	86/1162 (7.4)	6/2037 (0.29)	<0.001

HIV, Human immunodeficiency Virus; HBV, Hepatitis B Virus; HCV, Hepatitis C Virus; PWID, Person who injects drugs; MSM, Men who have sex with men; Detention, Admission to a lock up, prison or psychiatric facility; STI, sexually transmitted infection; IRM, Higher risk mother; IRB, Increased risk behaviour.

**FIGURE 2:**
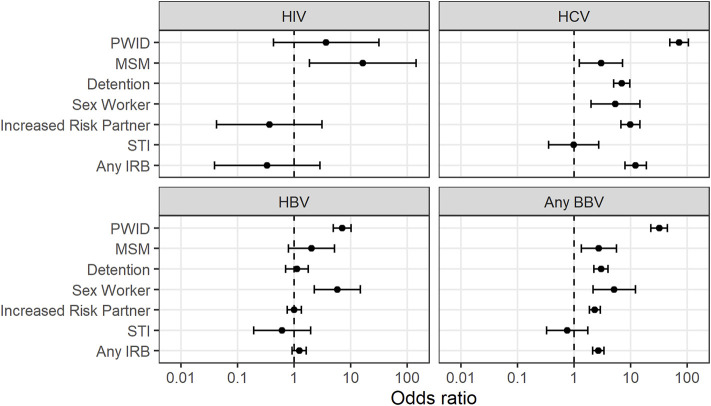
Association between increased risk behaviours and bloodborne virus exposure 7. PWID = Person who injects drugs. MSM = Men who have sex with men. STI = Sexually transmitted infection. IRB = Increased risk behaviour. BBV = blood-borne virus (HIV, HCV or HBV).

A history of a sexually transmitted infection such as syphilis, gonorrhoea or herpes was not associated with an increased prevalence of HIV, HBV or HCV (Figure 2, Supplementary Table S8).

“Any IRB” was associated with an increased prevalence of HCV (OR 12.7) but not HIV or HBV in potential organ donors (Table 4).

**TABLE 4:** Predictive performance of increased risk behaviours.

Virus	Sensitivity (%)	Specificity (%)	PPV (%)	OR	(95% CI)		p
HIV	17	62	0	0.33	0.00	2.15	0.420
HBV	47	63	1	1.47	0.74	2.92	0.143
HCV	88	65	11	13.80	8.74	21.8	<0.001
Any Active BBV	71	64	6	4.21	2.76	6.42	<0.001
Any BBV	60	65	15	2.75	2.19	3.46	<0.001

PPV, Positive predictive value; OR, Odds ratio. HIV- Human immunodeficiency Virus. HCV, Hepatitis C Virus. HBV, Hepatis B Virus. BBV, bloodborne virus.

In this study, “Any IRB” had only modest sensitivity and positive predictive power. One in every five potential donors with a BBV did not have any IRB identified by the BRAQ. Furthermore only 1 in every 8 patients identified as being IVRD had evidence of exposure to a BBV.

## Discussion

This is the first study to describe the prevalence of BBVs and IRBs amongst a national cohort of persons who have commenced workup for deceased organ donation within Australia.

The study has shown that potential organ donors in Australia have a higher prevalence of HCV, but similar rates of HIV and HBV when compared to the general population (18, 19).

Whilst a reported history of any IRB was common and associated with exposure to HCV, it was not associated with exposure to HBV or HIV.

### Blood-Borne Virus Exposure Prevalence

The significantly higher prevalence of HCV exposure seen in this study, when compared with the broader Australian population, is likely to derive from the over-representation of risk behaviour within the organ donor pool (4.9% vs 2.3%, *p* < 0.001).

Our study demonstrated that 5.2% of potential donors had a history of IDU, in contrast to 1.5% in the broader Australian population (23). IDU is the primary risk factor for HCV infection. The prevalence of HCV in PWID in Australia has been estimated to be 49% (18).

Our findings are consistent with this pattern of illness burden, with 62% of HCV exposed potential organ donors having a history of IDU and a 59% prevalence of HCV exposure in PWID.

Compared with international potential organ donor populations, Australia has similar rates of HCV when compared to US and Canada (4.98% vs 5.14% & 10.34%) and generally similar rates of HIV (0.16%) compared to with United States, Canada and the United Kingdom (0.21%, 0.00% and 0.06%) (24–26).

In contrast, our study showed higher rates of HBV when compared to potential organ donor cohorts in other nations (24–26). This is likely due to the higher overall prevalence of HBV within Australia, when compared to the UK and the US (27) (HBcAb Prevalence: 6.9%, vs 3.8% and 5.4%) (27, 28). Our reported prevalence of both active and inactive HBV in potential organ donors were similar to those published in a previous Australian national serosurvey (active HBV 0.9% and inactive HBV 4.95% in our study, versus HbsAg 0.8% and HBcAb 6.9% overall in Australia) (28).

Ninety-four percent of organ donors in Australia are adults, and newly acquired HBV infection in adulthood is uncommon. In Australia the majority of HBV infections are acquired during childhood, and occur most commonly in migrants from nations with higher endemicity (18). The attributable burden of disease associated with IRBs is thought to be only modest (PWID making up 5.7% and MSM making up 4.5% of those with HBV in Australia) (18). It is therefore unsurprising that the majority of in-active HBV infections in our study, occurred in individuals with no history of IRBs, and the ability of IRBs to predict acute HBV was poor.

### IRB Prevalence

Overall, IRBs appeared more common than previous estimates (3). However, direct comparisons with other studies are difficult due to variations in recency of exposure criteria required in differing international jurisdictions.

Our study reported that potential donors with IRBs were significantly younger than those without, and this finding is consistent with other studies of IVRDs and is an important fact as transplanted organs from younger donors have superior outcomes (8).

Our study showed an association between the presence of IRBs and a lower likelihood of progression to donation surgery. Future studies should better define the relationship between IVRD designation and organ utilisation in Australia.

### Virus Prevalence in IRB Cohorts

Overall, the prevalence of BBV exposures were similar to those reported in community-based cohorts who seemingly engage in the same IRBs (Supplementary Tables S9–S11). We differ in reporting lower rates of HCV in those with increased risk sexual partners (OR 0.47, *p* = 0.01), and those with a history of detention (OR 0.39, *p* < 0.001), and lower rates of active HBV in those with a history of detention (OR 0.3, *p* = 0.03) (see Supplementary Material for full analysis).

We report a strikingly high incidence of inactive HBV in potential donors who have had sex in exchange for money or drugs, when compared with HBcAB prevalence in community cohorts of predominantly commercial sex workers (7) (OR 16, *p* < 0.001). However in our series, sex work was not associated with active HBV. It may be that potential organ donors with these reported IRBs may represent a more culturally diverse cohort, a cohort with a higher number of migrant workers (29), or a higher proportion of sex-workers from the unregulated sector-any of which may be less represented in community cohort studies.

Exploration of the exact reasons for these differences in prevalence is beyond the scope of this study, but the finding provides a cautionary note when inferring risks of disease transmission from community-based cohorts.

Our study did not demonstrate an association between sexually transmitted infections and an increased prevalence of any of the BBVs. It is noteworthy that U.S. Department of Health and Human Services has recently removed this risk behaviour from their assessment of BBV transmission risk in organ donors (30). Our study would support a similar removal of this risk criteria within the Australian context.

### Implications

In this study, the presence of one or more IRBs was predictive of a higher prevalence of HCV, but not of HBV or HIV in potential organ donors. In an attempt to improve sensitivity, authorities have recently introduced a new, locally modified, IRB questionnaire, which has undergone cognitive evaluation overseas (31).

For HCV, where IRB screening is predictive, the clinical ramifications of unexpected donor-derived HCV infection are rapidly diminishing. Direct acting antivirals are well tolerated and successfully cure HCV in solid organ transplant recipients (32–35). The majority of patients with HBV or HIV did not have elicited IRBs.

This leads one to question the value of existing IRB screening. Routine donor NAT screening has shortened the diagnostic window considerably, and none of the IRBs sufficiently predict window period infection because it is uncommon even for the highest risk IRB [Death with a history of IVDU: Risk of undetected infection estimated as ∼1:50,000 for HIV, ∼1:2000 for HBsAg, ∼1:450 for HCV (7)].

If there is jurisdictional agreement to routinely use IVRD donors with negative NAT BBV tests, the more logical approach seems to be undertaking NAT in all recipients so that in the uncommon event of donor derived BBV infection it is detected and able to be treated before there are clinical ramifications. This approach has recently been adopted in the United States (30). The acceptability of such an approach within the broader Australian transplant community remains unknown.

Our study shows the prevalence of BBVs for some IVRD cohorts may be significantly different from previous estimations (7). It may not be appropriate to extrapolate prevalence rates from other cohorts to Australian donors, and inferences of residual risk may be affected. Further studies are required.

### Limitations

Given the rarity of window-period unexpected disease transmission from IVRDs and the overall small number of donors within Australia, it was not practical to design an appropriately powered study to assess the predictive power of IRBs on actual unexpected transmissions. Instead, a surrogate measure, the ability to predict established infection was used. It is possible, although though we feel unlikely, that IRBs may better predict very recent infection over established infection.

We adopted a conservative definition of exposure, and this may lead to overestimation of the prevalence of BBV in potential organ donors.

Several IRBs were rarely reported, and HIV had low prevalence. The study was, therefore, underpowered to reveal a significantly increased viral prevalence in sex-workers or in children of IRB mothers, and was underpowered to identify individual IRBs associated with HIV infection. Despite this, the study did demonstrate statistically significant correlation between sex work and exposure to HBV, and was adequately powered to detect higher prevalence of HIV in the composite IVRD cohort (OR 10 threshold).

Our study examined IRBs during a finite period of time preceding commencement of workup for organ donation rather than a history of having ever undertaken IRBs, and this may have affected the concordance with population studies. Additionally, in 2019 the TSANZ revised IRB exposure windows from “the last 12 months” to “the last 10 weeks”. This will in effect reduce the fraction of potential donors classified at IVRD (1). It is therefore likely that our study would have a higher rate of IVRD designation compared to a future case series.

Whilst not consistent with national guidelines, some potential organ donors are rejected prior to commencement of formal donation workup, either through self-censoring by the referring clinician, or based on cursory assessment by a donation service. The later having previously been documented within the local context (14). This may reduce the assessed predictive power or IRBs. Conversely, our findings of viral prevalence in potential donors are more likely to be more indicative, when compared to series where individual who do not procede to donation are excluded (36).

Caution should be applied when extrapolating our findings to other jurisdictions or differing populations. Our study examined non-self-reported behaviours, in a potential organ donor cohort in Australia. These behaviours may have differing predictive performance when self-reported (e.g., blood donors) or in settings with higher community prevalence, or in countries where the BBV burden is distributed differently according to specific IRBs.

### Conclusion

This study demonstrated that Australian potential organ donors had significantly higher rates of HCV and similar rates of HBV and HIV when compared to the broader population. Currently utilised risk behaviour assessment questionnaires were only moderately predictive of exposure or active infection with a blood borne virus. The utility of behavioural questionnaires in stratifying the risk of unexpected disease transmission may not provide the reassurance clinicians are seeking. Eliciting IRBs may be a redundant practice if organs from NAT negative IVRDs are routinely utilised and early BBV screening is performed in all recipients.

## Data Availability

The data analyzed in this study is subject to the following licenses/restrictions: Individual Australian States and Territories retain governance over data in the DonateLife Electronic Donor Record (EDR). Requests to access these datasets should be directed to Australian Organ and Tissue Authority, enquiries@donatelife.gov.au.

## Abbreviations

BBV, blood-borne virus; BRAQ, behavioural risk assessment questionnaire; EDR, electronic donor record; HBcAb, hepatitis B core antibody; HBsAb, hepatitis B surface antibody; HBsAg, hepatitis B surface antigen; HBV, hepatitis B virus; HCV, hepatitis C virus; HIV, human immunodeficiency virus; HIV Ag/Ab, HIV antigen/antibody combination test; IDU, injecting drug use; IRB, increased viral risk behaviour; IVRD, increased viral risk donor; MSM, men who have sex with men; NAT, nucleic acid test; PWID, person who injects drugs; TSANZ, transplantation society of Australia and New Zealand; US, United States; UK, United Kingdom.

## References

[1] The Transplantation Society of Australia and New Zealand. Clinical Guidelines for Organ Transplantation from Deceased Donors. Canberra (2020). Report No.: 1.4 Contract No.: 1.4. Australian Government. https://tsanz.com.au/storage/documents/TSANZ_Clinical_Guidelines_Version-15_29042021.pdf .

[2] Noble M Treadwell JR. Solid Organ Transplantation and the Probability of Transmitting HIV, HBV, or HCV; a Systematic Review to Support an Evidence-Based Guideline. Atlanta: ECRI Evidence-based Practice Center (2010). https://stacks.cdc.gov/view/cdc/12164

[3] Volk ML Wilk AR Wolfe C Kaul DR. The "PHS Increased Risk" Label Is Associated with Nonutilization of Hundreds of Organs Per Year. Transplantation (2017) 101(7):1666–9. 10.1097/tp.0000000000001673 28196048

[4] Sapiano MRP Jones JM Bowman J Levi ME Basavaraju SV. Impact of US Public Health Service Increased Risk Deceased Donor Designation on Organ Utilization. Am J Transpl (2019) 19(9):2560–9. 10.1111/ajt.15388 PMC6864734 30959569

[5] Irwin L Kotton CN Elias N Palafox J Basler D Shao SH Utilization of Increased Risk for Transmission of Infectious Disease Donor Organs in Solid Organ Transplantation: Retrospective Analysis of Disease Transmission and Safety. Transpl Infect Dis (2017) 19(6):e12791. 10.1111/tid.12791 28994164

[6] Kaul DR Tlusty SM Michaels MG Limaye AP Wolfe CR. Donor-derived Hepatitis C in the Era of Increasing Intravenous Drug Use: A Report of the Disease Transmission Advisory Committee. Clin Transpl (2018) 32(10):e13370. 10.1111/ctr.13370 30080289

[7] Waller KM De La Mata NL Kelly PJ Ramachandran V Rawlinson WD Wyburn KR Residual Risk of Infection with Blood‐borne Viruses in Potential Organ Donors at Increased Risk of Infection: Systematic Review and Meta‐Analysis. Med J Aust (2019) 211(9):414–20. 10.5694/mja2.50315 31489635

[8] Hwang CS Gattineni J MacConmara M. Utilizing Increased Risk for Disease Transmission (IRD) Kidneys for Pediatric Renal Transplant Recipients. Pediatr Nephrol (2019) 34(10):1743–51. 10.1007/s00467-019-04276-w 31243535

[9] Trotter PB Summers DM Robb M Hulme W Ushiro-Lumb I Watson CJE Deceased Organ Donors with a History of Increased Risk Behavior for the Transmission of Blood-Borne Viral Infection. Transplantation (2017) 101(7):1679–89. 10.1097/tp.0000000000001727 28291157

[10] Croome KP Lee DD Pungpapong S Keaveny AP Taner CB. What Are the Outcomes of Declining a Public Health Service Increased Risk Liver Donor for Patients on the Liver Transplant Waiting List? Liver Transpl (2018) 24(4):497–504. 10.1002/lt.25009 29341398

[11] Fleetwood VA Lusciks J Poirier J Hertl M Chan EY. Utilization of Public Health Service Increased Risk Donors Yields Equivalent Outcomes in Liver Transplantation. J Transpl (2016) 2016:9658904. 10.1155/2016/9658904 PMC5061985 27777790

[12] Limkemann AJ Wolfe L Sharma A King AL Behnke M Shah MJ Outcomes of Kidney Transplants and Risk of Infection Transmission from Increased Infectious Risk Donors. Clin Transpl (2016) 30(8):886–93. 10.1111/ctr.12761 27146714

[13] Okoh AK Chan O Schultheis M Gupta M Shah A Gold J Association between Increased-Risk Donor Social Behaviors and Recipient Outcomes after Heart Transplantation. Clin Transpl (2020) 34(3):e13787. 10.1111/ctr.13787 31961010

[14] Waller KMJ De La Mata NL Rosales BM Hedley JA Kelly PJ Thomson IK Characteristics and Donation Outcomes of Potential Organ Donors Perceived to Be at Increased Risk for Blood Borne Virus Transmission: an Australian Cohort Study 2010-2018. Transplantation (2022) 106:348. 10.1097/TP.0000000000003715 33988336

[15] White SL Rawlinson W Boan P Sheppeard V Wong G Waller K Infectious Disease Transmission in Solid Organ Transplantation: Donor Evaluation, Recipient Risk, and Outcomes of Transmission. Transplant Direct (2019) 5(1):e416. 10.1097/txd.0000000000000852 30656214 PMC6324914

[16] Australian Bureau of Statistics. 4102.0-Australian Social Trends. Canberra, Australia: Australian Bureau of Statistics (2013).

[17] Australian Bureau of Statistics. Net Overseas Migration, Arrivals and Departures, State/territory, Major Groupings and Visa - Calendar Years, 2004 to 2019. Canberra, Australia: Australian Bureau of Statistics (2020).

[18] The Kirby Institute. HIV, Viral Hepatitis and Sexually Transmissible Infections in Australia: Annual Surveillance Report 2017. Sydney: The Kirby Institute, The University of New South Wales (2017).

[19] Amin J Gidding H Gilbert G Backhouse J Kaldor J Dore G Hepatitis C Prevalence-Aa Nationwide Serosurvey. Commun Dis Intell Q Rep (2004) 28(4):517–21. 15745402 10.33321/cdi.2004.28.60

[20] Authority AGOaT. About Us (2020). Available from: https://donatelife.gov.au/about-us .

[21] Yao F Seed C Farrugia A Morgan D Wood D Zheng M-H. Comparison of the Risk of Viral Infection between the Living and Nonliving Musculoskeletal Tissue Donors in Australia. Transpl Int (2008) 21(10):936–41. 10.1111/j.1432-2277.2008.00703.x 18537922

[22] DeVita MA Brooks MM Zawistowski C Rudich S Daly B Chaitin E. Donors after Cardiac Death: Validation of Identification Criteria (DVIC) Study for Predictors of Rapid Death. Am J Transpl (2008) 8(2):432–41. 10.1111/j.1600-6143.2007.02087.x 18190657

[23] Australian Institute of H, Welfare. National Drug Strategy Household Survey 2019. Canberra, Australia: Australian Institute of Health and Welfare (2020).

[24] Davison KL Ushiro‐Lumb I Lawrance M Trotter P Powell JJ Brailsford SR. Infections and Associated Behaviors Among Deceased Organ Donors: Informing the Assessment of Risk. Transpl Infect Dis (2019) 21(2):e13055. 10.1111/tid.13055 30693636

[25] Ellingson K Seem D Nowicki M Strong DM Kuehnert MJ. Estimated Risk of Human Immunodeficiency Virus and Hepatitis C Virus Infection Among Potential Organ Donors from 17 Organ Procurement Organizations in the United States. Am J Transpl (2011) 11(6):1201–8. 10.1111/j.1600-6143.2011.03518.x 21645253

[26] Zahariadis G Plitt SS O'Brien S Yi Q-L Fan W Preiksaitis JK. Prevalence and Estimated Incidence of Blood-Borne Viral Pathogen Infection in Organ and Tissue Donors from Northern Alberta. Am J Transpl (2007) 7(1):226–34. 10.1111/j.1600-6143.2006.01607.x 17109730

[27] Custer B Sullivan SD Hazlet TK Iloeje U Veenstra DL Kowdley KV. Global Epidemiology of Hepatitis B Virus. J Clin Gastroenterol (2004) 38(10 Suppl. 3):S158–S168. 10.1097/00004836-200411003-00008 15602165

[28] O'Sullivan BG Gidding HF Law M Kaldor JM Gilbert GL Dore GJ. Estimates of Chronic Hepatitis B Virus Infection in Australia, 2000. Aust N Z J Public Health (2004) 28(3):212–6. 10.1111/j.1467-842x.2004.tb00697.x 15707165

[29] Renshaw L Kim J Australian Institute of C Scarlet A. Migrant Sex Workers in Australia. Canberra: Australian Institute of Criminology (2015).

[30] Jones JM Kracalik I Levi ME Bowman JS 3rd Berger JJ Bixler D Assessing Solid Organ Donors and Monitoring Transplant Recipients for Human Immunodeficiency Virus, Hepatitis B Virus, and Hepatitis C Virus Infection - U.S. Public Health Service Guideline, 2020. MMWR Recomm Rep (2020) 69(4):1–16. 10.15585/mmwr.rr6904a1 PMC7337549 32584804

[31] Willson S Miller K Seem D Kuehnert MJ. Cognitive Evaluation of the AABB Uniform Donor History Questionnaire. Transfusion (2016) 56(6 Pt 2):1662–7. 10.1111/trf.13587 27060456

[32] Cotter TG Paul S Sandıkçı B Couri T Bodzin AS Little EC Increasing Utilization and Excellent Initial Outcomes Following Liver Transplant of Hepatitis C Virus (HCV)-Viremic Donors into HCV-Negative Recipients: Outcomes Following Liver Transplant of HCV-Viremic Donors. Hepatology (2019) 69(6):2381–95. 10.1002/hep.30540 30706517

[33] Woolley AE Singh SK Goldberg HJ Mallidi HR Givertz MM Mehra MR Heart and Lung Transplants from HCV-Infected Donors to Uninfected Recipients. N Engl J Med (2019) 380(17):1606–17. 10.1056/nejmoa1812406 30946553 PMC7369135

[34] Reese PP Abt PL Blumberg EA Van Deerlin VM Bloom RD Potluri VS Twelve-Month Outcomes after Transplant of Hepatitis C-Infected Kidneys into Uninfected Recipients: A Single-Group Trial. Ann Intern Med (2018) 169(5):273–81. 10.7326/m18-0749 30083748

[35] Gasink LB Blumberg EA Localio AR Desai SS Israni AK Lautenbach E. Hepatitis C Virus Seropositivity in Organ Donors and Survival in Heart Transplant Recipients. JAMA (2006) 296(15):1843–50. 10.1001/jama.296.15.1843 17047214

[36] Abara WE Collier MG Moorman A Bixler D Jones J Annambhotla P Characteristics of Deceased Solid Organ Donors and Screening Results for Hepatitis B, C, and Human Immunodeficiency Viruses - United States, 2010-2017. MMWR Morb Mortal Wkly Rep (2019) 68(3):61–6. 10.15585/mmwr.mm6803a2 30677008 PMC6348762

