# Supplementary material for: Prevalence of Blood-Borne Viruses and Predictors of Risk in Potential Organ Donors in Australia
Source: Transpl Int. 2022 May 3;35:10395. doi: 10.3389/ti.2022.10395 (PMC9110643; doi:10.3389/ti.2022.10395)
Supplement: Supplementary file 1 [file DataSheet1.PDF]

Supplementary Digital Content

Table of Contents

Supplementary Digital Content **1**

Table of Contents **1**

1 Sample Size Calculation **2**

Introduction **2**

Table S1: Prevalence of Blood Borne Viruses 2

Methods & Rationale **2**

Selection of a population to model prevalence 2

Selection of a significant Odds Ratio 3

Selection of Experimental to Control ratio 3

Table S2: Case Control Ratios 4

Estimation of the case series size 4

Results **4**

Table S3: Sample Size Estimation for HIV, HBV and HCV Viruses* 5

Discussion 5

Conclusion **5**

References **6**

2. Haemodilution Criteria **7**

Figure S1: Haemodilution Criteria 7

3. Definitions of BBV Exposure **8**

Table S4: Definitions of BBV Exposure 8

4. TSANZ Increased Viral Risk Donor Criteria **9**

Table S5: IVRD Criteria 9

5. Selected questions from the DonateLife behavioural risk assessment questionnaire **10**

Table S6: DonateLife Behavioural Risk Assessment Questionnaire 10

6. Mapping of the behavioural risk assessment questionnaire to TSANZ IVRD criteria **11**

Table S7: Risk: Questionnaire Mapping 11

7. Predictors of blood borne virus analysis **12**

Table S8: Predictors of BBV 12

8. Comparison of prevalence with Waller meta-analysis **13**

Table S9 13

Table S10 15

Table S11 16

Figure S2 17

1 Sample Size Calculation

Introduction

The prevalence of blood borne viruses has not been previously described in potential organ donors in Australia. In order to assess the minimum required sample size for the reliable detection of clinically meaningful differences in prevalence for various risk factors, prevalence of blood borne virus in various surrogate populations were reviewed (Table 1).

Table S1: Prevalence of Blood Borne Viruses

	Potential Organ Donors			Other Populations		
	Standard Risk Organ Donors	All Organ Donors	All Organ Donors	National Prevalence	First Time Blood Donors	Deceased MSK Donors
**Country**	US(1)	Canada(2)	UK(3)	Australia(4)	Australia(5)	Australia(6)
**Prevalence**	%	%	%	%	%	%
**HIV**	0.1	0.00	0.10	0.01	0.01	0.07
**HBV**	n/a	n/a	0.94	0.29	0.14	0.88
**HCV**	3.45	0.23	0.06	0.12	0.22	0.27

Methods & Rationale

Sample size calculation were undertaken using STATA IC 15.1, using the "power twoproportions" function. The significance level was set to 0.05, with a power level of 0.80. Two tailed probabilities were calculated.

Selection of a population to model prevalence

We elected to use the prevalence of virus in Australian deceased Musculo-skeletal donors to model the probability of exposure amongst controls(6). Deceased musculoskeletal donors significantly overlap with solid organ deceased donors, with increased viral risk donors screened out due to TGA requirements.

Selection of a significant Odds Ratio

Nomination of a clinically significant odds ratio can be viewed through both the prism of strength of association between the presence of a risk factor and disease (eg through the use of Cohen's D), and the minimally discernible difference individuals are able to weigh.

Hill described the importance of strength of association when assessing the likelihood of causation between risk factors and disease in epidemiological studies. A number of authors argue that in epidemiologic studies an odd ratio of 3 supports at least a moderate strength of association. This is particularly the case when the prevalence is low (less than 10%)(7, 8).

However, risk is unlikely to be perceived psychometrically on a linear scale. Arguments have been made that risk is more likely quantified on a logarithmic scale. Similar to work by Wong and Baker who describe that pain was more appropriately defined on a 5-point scale (rather than 10), it has been argued that perceptible differences in risk occur between orders of magnitude. Furthermore at the extremes of risk, perception is further distorted, with the exaggerated -weighing of low and higher risk events (9, 10).

Based on the above, sample size was modelled for two differing odds ratios: 3 and 10.

Selection of Experimental to Control ratio

The ratio of cases to control for sample size estimation depends on the prevalence of each individual risk factor within the community. The prevalence of each risk factor has not been described within the potential organ donor community, so a battery of estimations has been performed for risk factor prevalence of 1%,3%, 5%,10%,30%. The relationship between risk factor prevalence and experimental control ratio is described in table 2.

Table S2: Case Control Ratios

Risk Factor Prevalence (%)	Experimental : Control Ratio
**1**	0.010
**3**	0.031
**5**	0.053
**10**	0.111
**30**	0.423

Estimation of the case series size

In 2019 there were 548 deceased organ donors. Simple extrapolation over a 6-year period (2014-2020) would produce an estimated case series of approximately 3288. Not all patients who commence work-up for organ donation, proceed to donation, so this calculation would likely underestimate total case series size.

Results

Estimated sample sizes have been calculated and plotted for each virus, based on prevalence previously described in Australian deceased musculoskeletal tissue donors (Table 3 and Figure 1). Calculations have been made for minimum total sample, for odds ratios that represent both moderate strength of association (OR3) and clinically perceptible quantum of change in risk (OR10), and a variety of different risk factor prevalence (Table 3).

Table S3: Sample Size Estimation for HIV, HBV and HCV Viruses*

Prevalence (%)	HIV: OR 10	HIV: OR 3	HBV: OR 10	HBV: OR 3	HCV: OR 10	HCV: OR 3
1	37,774	443,622	3,170	34,940	9,660	111,581
2	20,127	228,145	1,681	17,965	5,140	57,381
5	9,065	94,136	749	7,407	2,309	23,672
10	5,502	51,984	450	4,087	1,398	13,069
30	3,187	25,218	258	1,977	808	6,335

*Cells where estimated sample size is likely to exceed case series size are shaded dark grey.

Discussion

Due to the low prevalence of these viruses within the community, significant sample sizes will be required to demonstrate a significant difference for a number of risk factors, particularly those with low prevalence within the community. For HIV; the projected case serries size will unlikely be powered to detect differences for any risk factor an OR of 3, and only risk factors with a prevalence of 15% or above for an OR of 10. For HBV; the projected case serries size will likely be able to detect differences at an OR of 3 for risk factors with a broad variety of prevalence. However, it will not be powered to detect differences at an OR of 3 for factors with a prevalence less than 10%. For HCV, the projected sample size will be appropriately powered to determine OR effect sizes of 10 or more, in all risk-factors, except those with the lowest prevalence. It will not be powered to detect OR 3 differences for HCV.

Conclusion

It is anticipated that the proposed study will be appropriately powered to determined clinically meaningful differences in prevalence of HBV at a risk-factor prevalence of >=1%, and for HCV at a risk factor prevalence > 2%.
Unfortunately, the study is not powered to reliably identify differences in prevalence of HIV between risk cohorts in all, but the highest prevalence risk factors.

References

1. Ellingson K, Seem D, Nowicki M, Strong DM, Kuehnert MJ, Organ Procurement Organization Nucleic Acid Testing Yield Project T. Estimated risk of human immunodeficiency virus and hepatitis C virus infection among potential organ donors from 17 organ procurement organizations in the United States. Am J Transplant. 2011;11(6):1201-8.
2. Zahariadis G, Plitt SS, O'Brien S, Yi QL, Fan W, Preiksaitis JK. Prevalence and estimated incidence of blood-borne viral pathogen infection in organ and tissue donors from northern Alberta. Am J Transplant. 2007;7(1):226-34.
3. Davison KL, Ushiro-Lumb I, Lawrance M, Trotter P, Powell JJ, Brailsford SR. Infections and associated behaviors among deceased organ donors: Informing the assessment of risk. Transpl Infect Dis. 2019;21(2):e13055.
4. Institute K. HIV, viral hepatitis and sexually transmissible infections in Australia: annual surveillance report 2018. Kirby Institute, UNSW Sydney Sydney; 2019.
5. Yao F, Seed C, Farrugia A, Morgan D, Cordner S, Wood D, et al. The risk of HIV, HBV, HCV and HTLV infection among musculoskeletal tissue donors in Australia. Am J Transplant. 2007;7(12):2723-6.
6. Yao F, Seed C, Farrugia A, Morgan D, Wood D, Zheng MH. Comparison of the risk of viral infection between the living and nonliving musculoskeletal tissue donors in Australia. Transpl Int. 2008;21(10):936-41.
7. Chen H, Cohen P, Chen S. How Big is a Big Odds Ratio? Interpreting the Magnitudes of Odds Ratios in Epidemiological Studies. Communications in Statistics - Simulation and Computation. 2010;39(4):860-4.
8. Rosenthal JA. Qualitative Descriptors of Strength of Association and Effect Size. Journal of Social Service Research. 1996;21(4):37-59.
9. Calman SKC, Royston G. Personal paper: Risk language and dialects. BMJ. 1997;315(7113):939.
10. Kahneman D, Tversky A. Prospect Theory: An Analysis of Decision under Risk. Econometrica. 1979;47(2):263-91.

2. Haemodilution Criteria

The following table is based on guidance from AOTA, and defined the classification of a sample as haemodiluted.

Figure S1: Haemodilution Criteria

Haemodilution Criteria

Haemodilution is said to be present when:

Total volume of blood products transfused in the last 48hrs

Plus

Total volume of colloids infused in the last 48hrs

Plus

Total Volume of Crystalloids infused in the last hour

Is =>

Total Blood volume

OR

Total volume of colloids infused in the last 48hrs

Plus

Total Volume of Crystalloids infused in the last hour

Is =>

Total Plasma volume

Where:

TPV = Weight (kg) / 0.025

TBV = Weight (kg) / 0.015

3. Definitions of BBV Exposure

Table S4: Definitions of BBV Exposure

	Disease		
**Classification**	**HBV**	**HCV**	**HIV**
**Positive**			
**Criteria: Any one of the following tests positive**	Anti-HBcAb<br>HBcAB IgM<br>HBsAg<br>NAT HBV	Anti-HCV<br>NAT HCV	Anti-HIV I/II<br>NAT HIV
**Negative**			
**Criteria: 1) At least one of the following tests performed**	NAT HIV+HBV+HCV<br>Anti-HBcAb<br>HBcAB IgM<br>HBsAg<br>NAT HBV	NAT HIV+HBV+HCV<br>Anti-HCV<br>NAT HCV	NAT HIV+HBV+HCV<br>Anti-HIV I/II<br>NAT HIV
**Criteria 2: Of those tests performed, none of the following are positive, and at least one is negative.**	Anti-HBcAb<br>HBcAB IgM<br>HBsAg<br>NAT HBV	Anti-HCV<br>NAT HCV	Anti-HIV I/II<br>NAT HIV
**Unknown Status**			
**Criteria: None of the following tests have been performed or tests have been performed on a haemodiluted sample.**	NAT HIV/HBV/HCV Triplex<br>Anti-HBcAb<br>HBcAB IgM<br>HBsAg<br>NAT HBV	NAT HIV/HBV/HCV Triplex<br>Anti-HCV<br>NAT HCV	NAT HIV/HBV/HCV Triplex<br>Anti-HIV I/II<br>NAT HIV

4. TSANZ Increased Viral Risk Donor Criteria

Table S5: IVRD Criteria

**IVRD Criteria**
People known or highly suspected to have HIV, HBV and/or HCV infection
People who have injected drugs by intravenous, intramuscular, or subcutaneous route for non-medical reasons in the preceding 10 weeks*
MSM in the preceding 10 weeks
People who have been in lockup, jail, prison, or a juvenile correctional facility for more than 72 consecutive hours in the preceding 10 weeks
People who have had sex in exchange for money or drugs in the preceding 10 weeks
People who have had sex with a person in any of the above groups in the preceding 10 weeks
People who have been newly diagnosed with, or have been treated for, syphilis, gonorrhoea, chlamydia, or genital ulcers in the preceding 10 weeks
A child who is 18 months old or younger and born to a mother known to be infected with, or at increased risk for, HIV, HBV or HCV infection
A child who has been breastfed within the preceding 6 months, and the mother is known to be infected with, or at increased risk for, HIV, HBV or HCV infection
When a deceased potential organ donor's medical/behavioural history cannot be obtained, or risk factors cannot be determined
When a deceased potential organ donor's blood specimen is haemodiluted so that testing for HIV, HBV, and HCV infection is less reliable

* 10 weeks represents the longest serological window for detection of any of these three bloods borne viruses (i.e. HCV, which has a serological window of ~70 days).

5. Selected questions from the DonateLife behavioural risk assessment questionnaire

Table S6: DonateLife Behavioural Risk Assessment Questionnaire

Question Number	Question
**20a**	To the best of your knowledge has he/she ever injected, inhaled or snorted drugs, even once, which were NOT prescribed by a doctor or dentist?
**20b**	To the best of your knowledge has he/she ever had a test which showed he/she had Hepatitis B, C, HIV or HTLV?
**25**	Within the last 12 months has he/she been in a lock up, prison or psychiatric facility?
**27a**	MALES – Within the last 12 months has he had male to male sex?
**27b**	FEMALES – Within the last 12 months has she had sex with a bisexual man?
**28**	Within the last 12 months has he/she been a sex worker (e.g. received payment in money, gifts or drugs)?
**29**	Within the last 12 months has he/she had sex with a sex worker?
**30**	Within the last 12 months has he/she been sexually active with someone known or suspected to have HIV or Hepatitis B or C?
**31**	Within the last 12 months has he/she had sex with someone who's been in prison?
**32**	Within the last 12 months has he/she had sex with an injecting drug user?
**34**	Within the last 12 months has he/she been diagnosed with or treated for a sexually transmitted disease such as syphilis, gonorrhoea or herpes?

6. Mapping of the behavioural risk assessment questionnaire to TSANZ IVRD criteria

Table S7: Risk: Questionnaire Mapping

Behavioural Risk	Behavioural risk assessment questionnaire Mapping
**People who have injected drugs by intravenous, intramuscular, or subcutaneous route for non-medical reasons**	Q20a AND age >2
**Men who have sex with men**	Q27a AND age >2
**People who have been in lockup, jail, prison, or a juvenile correctional facility for more than 72 consecutive hours**	Q25 AND age >2
**People who have had sex in exchange for money or drugs**	Q28 AND age >2
**People who have had sex with a person in any of the above groups**	(Q27b OR Q29 OR q30 OR q31 OR q32) AND age >2
**People who have been newly diagnosed with, or have been treated for, syphilis, gonorrhoea, chlamydia, or genital ulcers**	Q34 AND age >2
**A child who is 18 months old or younger and born to a mother known to be infected with, or at increased risk for, HIV, HBV or HCV infection**	Age<2 AND (Q20a OR q20b OR q25 OR q27a OR 28 OR q27b OR q30, q31 OR q32 OR q34
**A child who has been breastfed within the preceding 6 months, and the mother is known to be infected with, or at increased risk for, HIV, HBV or HCV infection**	Age<5 AND (Q20a, q20b, q25 OR q27a OR 28 OR q27b OR q30 OR q31OR q32 OR q34)

Note:

1. TSANZ criteria often include a proximity of exposure criteria. This was not included for recreational drug use.
2. The BRAQ questions do not always perfectly align with TSANZ criteria (Eg. TSANZ: "People who have been in lockup, jail, prison, or a juvenile correctional facility for more than 72 consecutive hours", VS BRAQ "Within the last 12 months has he/she been in a lock up, prison ***<u>or psychiatric facility</u>***?"
3. For paediatric donors, given the scope of the dataset release, a number of assumptions have been made to map the TSANZ criteria to the EDR data. The following assumptions were made to map criteria.
    a. All IRB reported in a child's BRAQ were presumed to be behaviours of the mother.
    b. A cut-off age of less than 2years was substituted for less than 18months in the TSANZ criteria.
    c. For the purposes of analysis, all children less than 5 years of age were assumed to have been ceased breast-feeding less than 6 months ago. This is likely to be overly inclusive.

7. Predictors of blood borne virus analysis

The odds ratios resulting from the univariate logistic regression models are shown below. Note the implausible odds ratios with infinitely wide confidence intervals for HIV and Detention, Sex Worker and STI - this is because no HIV positive cases had these exposures.

Table S8: Predictors of BBV

Exposure	HIV	HCV	HBV	Any BBV
PWID	3.7 (0.43, 32)	72 (50, 105)	7.1 (4.9, 10)	32 (23, 45)
MSM	16 (1.9, 143)	3.0 (1.3, 7.2)	2.0 (0.79, 5.2)	2.8 (1.4, 5.6)
Detention	0.00 (0.00, Inf)	7.0 (5.1, 9.7)	1.1 (0.71, 1.8)	3.0 (2.3, 4.0)
Sex Worker	0.00 (0.00, Inf)	5.4 (2.0, 15)	5.8 (2.3, 15)	5.2 (2.2, 12)
Increased Risk Partner	0.37 (0.04, 3.1)	10.0 (6.7, 15)	1.0 (0.76, 1.3)	2.3 (1.9, 2.9)
STI	0.00 (0.00, Inf)	0.99 (0.36, 2.7)	0.61 (0.19, 2.0)	0.75 (0.33, 1.7)
Any IRB	0.33 (0.04, 2.9)	12 (8.0, 19)	1.2 (0.93, 1.6)	2.7 (2.2, 3.4)

Results of this analysis are graphed in Figure 2: Association between increased risk behaviours and bloodborne virus exposure.

8. Comparison of prevalence with Waller meta-analysis

To compare prevalence estimates between the serosurvey results and the Waller et al 2017 meta-analysis, we calculated log odds for prevalence for each study, along with associated standard errors.

For the serosurvey, the number of infected and exposed was known for each disease and each exposure, so log odds and standard errors could be calculated using the usual formulas. A zero-cell correction of 0.5 was added to each cell of the 2x2 table to avoid dividing or taking logs of zero. See e.g.: https://handbook-5-1.cochrane.org/chapter_16/16_9_2_studies_with_zero_cell_counts.htm; or Borenstein et al [find reference].

For the meta-analysis, the log odds were calculated from the reported prevalence. The standard errors were calculated from the reported 95% confidence intervals, assuming they were derived from back-transforming calculations on a log odds scale. For cases where the lower confidence limit was 0.0%, the lower confidence limit was estimated using (prevalence^2 / upper_confidence_limit).

The odds ratio for difference in prevalence was calculated using the difference in log odds between the two studies. The associated standard error was calculated using the root-sum-square of the individual studies' standard errors.

The table below shows prevalence estimates from our study:

Table S9

exposure_name	n_infected	n_exposed	prevalence	log_odds	log_odds_se
HIV					
PWID	1	188	0.005	?4.83	0.82
MSM	1	45	0.022	?3.39	0.83
Detention	0	340	0.000	?6.52	1.42
Sex Worker	0	23	0.000	?3.85	1.43
Increased Risk Partner	1	1290	0.001	?6.76	0.82
STI	0	81	0.000	?5.09	1.42
Any IRB	1	1367	0.001	?6.81	0.82
HCV					
PWID	113	190	0.595	0.38	0.15
MSM	6	45	0.133	?1.80	0.42

exposure_name	n_infected	n_exposed	prevalence	log_odds	log_odds_se
Detention	68	340	0.200	?1.38	0.14
Sex Worker	5	23	0.217	?1.21	0.49
Increased Risk Partner	151	1292	0.117	?2.02	0.09
STI	4	81	0.049	?2.85	0.48
Any IRB	158	1369	0.115	?2.03	0.08
HBV					
PWID	49	188	0.261	?1.04	0.17
MSM	5	45	0.111	?2.00	0.45
Detention	22	341	0.065	?2.65	0.22
Sex Worker	6	23	0.261	?0.99	0.46
Increased Risk Partner	76	1291	0.059	?2.77	0.12
STI	3	81	0.037	?3.11	0.55
Any IRB	90	1367	0.066	?2.65	0.11
Any BBV					
PWID	130	190	0.684	0.77	0.16
MSM	10	45	0.222	?1.22	0.35
Detention	73	341	0.214	?1.30	0.13
Sex Worker	8	23	0.348	?0.60	0.43
Increased Risk Partner	188	1293	0.145	?1.77	0.08
STI	6	81	0.074	?2.45	0.41
Any IRB	207	1370	0.151	?1.72	0.08

The table below shows prevalence estimates from the Waller meta-analysis:

Table S10

exposure_name	proportion	ci_lower	ci_upper	log_odds	log_odds_se
HIV - HIV					
PWID	0.007	0.001	0.014	?4.95	0.67
MSM	0.052	0.025	0.088	?2.90	0.32
Detention	0.002	0.001	0.005	?6.21	0.47
Sex Worker	0.002	0.001	0.007	?6.21	0.64
Increased Risk Partner	0.002	0.000	0.011	?6.21	0.87
HCV - HCV					
PWID	0.660	0.570	0.738	0.66	0.07
MSM	0.040	0.003	0.110	?3.18	0.92
Detention	0.389	0.254	0.533	?0.45	0.19
Sex Worker	0.180	0.005	0.490	?1.52	1.17
Increased Risk Partner	0.220	0.120	0.360	?1.27	0.28
HBV - HBSAG					
PWID	0.035	0.009	0.079	?3.32	0.55
MSM	0.015	0.001	0.042	?4.18	0.95
Detention	0.042	0.025	0.064	?3.13	0.24
HBV - HbCAb					
PWID	0.363	0.277	0.454	?0.56	0.13
MSM	0.096	0.032	0.189	?2.24	0.45
Detention	0.195	0.103	0.307	?1.42	0.28
Sex Worker	0.023	0.015	0.034	?3.75	0.21
Increased Risk Partner	0.008	0.002	0.022	?4.82	0.61

The table below shows odds ratios for the difference in prevalence between the two studies, along with associated 95% confidence intervals and p-values. **Odds ratios greater than 1 indicate higher prevalence in the serosurvey study than Waller et al 2019.**

Table S11

Disease	PWID	MSM	Detention	Sex Worker	Increased Risk Partner
HIV	1.1 (0.14, 9.1) p=0.905	0.61 (0.11, 3.5) p=0.584	0.73 (0.04, 14) p=0.835	11 (0.49, 228) p=0.131	0.58 (0.06, 6.0) p=0.649
HCV	0.75 (0.55, 1.0) p=0.081	3.9 (0.54, 29) p=0.175	0.39 (0.25, 0.62) p<0.001	1.4 (0.11, 16) p=0.811	0.47 (0.26, 0.84) p=0.010
Active HBV	1.5 (0.41, 5.2) p=0.559	0.72 (0.03, 21) p=0.849	0.30 (0.11, 0.86) p=0.025		
Non-active HBV	0.48 (0.31, 0.73) p<0.001	1.3 (0.36, 4.5) p=0.702	0.24 (0.11, 0.48) p<0.001	16 (5.9, 42) p<0.001	6.5 (1.9, 22) p=0.003

The figure below shows the same information graphically. Error bars show 95% confidence intervals for the odds ratios.

Figure S2

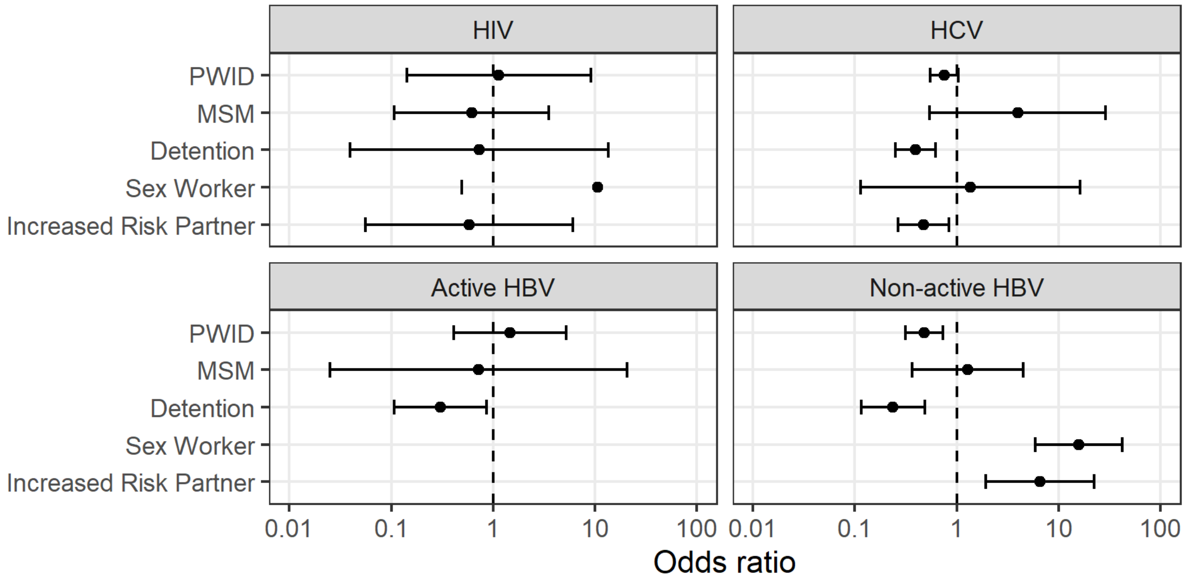

Note: Waller HBsAg compared with Active HBV. Wall HBcAb compared with InActive HBV.

Comparison Source:

Waller KM, De La Mata NL, Kelly PJ, et al. Residual risk of infection with blood-borne viruses in potential organ donors at increased risk of infection: systematic review and meta-analysis. *Med J Aust*. 2019;211(9):414-420. doi:10.5694/mja2.50315
